# Elaboration and Characterization of Electrodes from *Robinia pseudoacacia* and *Azadirachta indica* Charcoal Powder with Coconut Bio-Pitch as a Binder

**DOI:** 10.3390/ma17215156

**Published:** 2024-10-23

**Authors:** Epiphane Zingbe, Damgou Mani Kongnine, Bienvenu M. Agbomahena, Pali Kpelou, Essowè Mouzou

**Affiliations:** 1 Regional Center of Excellence for Electricity Control (CERME), University of Lome, Lome 01 BP 1515, Togo; 2 Faculty of Sciences, Department of Physics, Solar Energy Laboratory, University of Lome, Lome 01 BP 1515, Togo; 3 Laboratory of Electrical Engineering, Telecommunications and Applied Computing, University of Abomey Calavi UAC/EPAC, Abomey Calavi 229, Benin; 4 Faculty of Sciences, Department of Physics, Laboratory of Physics of Materials and Semiconductor Components, University of Lome, Lome 01 BP 1515, Togo

**Keywords:** bio-pitch, electrodes, mass loss, density, electrical resistivity, roughness

## Abstract

Carbon-based electrodes have recently been most widely used in P-MFC due to their desirable properties such as biocompatibility, chemical stability, affordable price, corrosion resistance, and ease of regeneration. In general, carbon-based electrodes, particularly graphite, are produced using a complex process based on petroleum derivatives at very high temperatures. This study aims to produce electrodes from bio-pitch and charcoal powder as an alternative to graphite electrodes. The carbons used to manufacture the electrodes were obtained by the carbonisation of *Robinia pseudoacacia* and *Azadirachta indica* wood. These carbons were pulverised, sieved to 50 µm, and used as the raw materials for electrode manufacturing. The binder used was bio-pitch derived from coconut shells as the raw materials. The density and coking value of the bio-pitch revealed its potential as a good alternative to coal-tar pitch for electrode manufacturing. The electrodes were made by mixing 66.50% of each carbon powder and 33.50% of bio-pitch. The resulting mixture was moulded into a cylindrical tube 8 mm in diameter and 80 mm in length. The raw electrodes obtained were subjected to heat treatment at 800 °C or 1000 °C in an inert medium. The electrical resistivity obtained by the four-point method showed that N1000 has an electrical resistivity at least five times lower than all the electrodes developed and two times higher than that of G. Fourier-transform infrared spectroscopy (FTIR) was used to determine the compositional features of the samples and their surface roughness was characterised by atomic force microscopy (AFM). Charge transfer was determined by electrical impedance spectroscopy (EIS). The FTIR of the electrodes showed that N1000 has a spectrum that is more similar to that of G compared to the others. The EIS showed the high ionic mobility of the ions and therefore that N1000 has a higher charge transfer compared to G and the others. AFM analysis revealed that N1000 had the highest surface roughness in this study.

## 1. Introduction

Over the last few decades, the crucial issues of the energy crisis and environmental pollution have become global challenges. The development of renewable energy sources for the energy transition and environmental protection is attracting sustained interest. Among the innovations, the plant-based microbial fuel cell (P-MFC) stands out because it exploits the electrochemical activity of microorganisms at the anode. These microorganisms oxidise the organic matter (substrate) releasing electrons which are then collected by the anode and sent to the external circuit. The renewable nature is due to the fact that the substrate is produced by photosynthesis using solar radiation [1]. P-MFC technology is unique in that it uses biocatalysts, eliminating the need for catalysts and chemical mediators. P-MFC technology has been linked to wastewater treatment [2], soil remediation [3], greenhouse gas emission reductions [4], and other applications. It can be combined with agricultural production without interfering with food production. PMFC technology is an alternative source of bioenergy for the future that is green, clean, renewable, sustainable, and much cheaper than any other form of bioenergy [5]. However, its low power density and comparatively high cost remain major challenges for the development of P-MFCs.

Recent research has explored various strategies such as the use of different plant species [6], growth media [7], and specific cell configurations [8] to improve the energy production of P-MFCs. The influence of carbon-based materials as electrodes for P-MFCs has been the focus of most studies. In the context of P-MFCs, both electrodes (anode and cathode) play crucial roles, but only the anode is responsible for bacterial growth, electron collection, and electron transfer to the cathode [9]. The anode of a P-MFC must have properties such as adequate roughness, excellent biological compatibility, a large surface area, high conductivity, chemical stability, and efficient electron transfer between the microorganisms and the anode surface [10]. The choice of materials used in the design of the anode is critical to the cost and performance of the P-MFC as demonstrated by Linghan Lan in 2019 [11]. Studies show several carbon-based materials, metallic materials, graphene, polymer composite based materials, composite materials using oxides such as TiO_2_, ZnO, MgO, etc. [12]. The literature reports that conventional carbon-based materials (graphite rod, carbon rod, carbon paper, graphite paper, graphite fabric, etc.), polymer composites used as electrodes for P-MFC have shown drawbacks such as cost problem, reduced surface area, low conductivity [12]. Metal electrodes have failed due to the problem of corrosion, which does not allow long-term stability and is therefore incompatible with bacterial growth [13]. The use of graphene is fairly expensive for electrode manufacture [13]. A recent study showed that graphene outperformed all conventional electrodes in terms of performance [14]. However, its toxicity to microorganisms is an obstacle [5]. Composite electrodes also exhibit oxygenated compounds on their surface, as evidenced by their Fourier transform infrared spectra [15]. To improve electron transfer and optimise microbial activities at the anode surface, significant modifications or designs of the electrode are required [9]. Although various efforts have been made to develop and modify electrode materials, very few environmentally friendly and non-toxic approaches have been tested. Research into carbon electrode materials is attracting particular attention due to their inherent advantages such as the abundance of raw materials and their cost effectiveness, environmental friendliness, and biocompatibility with microorganisms. Different methods are used to manufacture electrodes [16]. Activated carbons obtained as anodes derived from biomass are generally obtained by direct pyrolysis at high temperature with chemical activation leading to the formation of a porous carbon structure [17]. For the various activated carbons synthesised as anodes, the electrical resistivity ρ < 0. 2 Ω.cm [18]. When electrodes are made from powdered charcoal or coke using sugar syrup or coal tar as a binder, the electrical resistivity of the electrode drops to 10^−2^ Ω.cm when heat treated at temperatures above 900 °C [18]. This approach, although ingenious, is far from solving the problem due to the low electrical resistivity compared to industrial graphite whose resistivity is of the order of μΩ.m. Recently, Asim A. Yaqoob et al. developed graphene oxide (GO) anodes derived from biomass. They added metal oxides (ZnO and TiO_2_) as modifiers. Graphene oxides and composite electrodes based on graphene are limited in their application because of the problems mentioned above. Analysis of their infrared spectra shows the presence of oxygen compounds [15], which can cause passivation over time. Kumar Sonu et al. [19] developed a composite anode by inserting an ordinary carbon rod into the hollow of a carbonised corn cob. The use of this anode in P-MFC gives much higher power than with an ordinary carbon rod anode. In this work, an ordinary carbon rod of fossil origin is always used. Industrially, the anode is prepared using coal-tar coke, coal-tar pitch, or paraffin and nujol as a binder. The specifications of the electrodes, which are mainly based on the upper limits of sulphur and heavy metal content, density, molecular structure, and mechanical properties, do not comply with the needs of a green revolution [20]. Replacing petroleum coke with charcoal in the anode recipe would reduce fossil CO_2_ emissions [21]. Coutinho et al. [8] developed anodes by mixing charcoal and fossil pitch. These anodes were treated at 2700 °C. The electrical resistivity obtained by the authors is of the order of 10^−2^ Ω.m. However, the heat treatment temperature makes this process difficult to apply industrially.

The aim of this study is to develop carbon electrodes from *Robinia pseudoacacia* and *Azadirachta indica* charcoal and coconut shell bio-pitch. These three biomasses are locally available and replace polluting and environmentally harmful fossil fuels and rely on a locally applicable maximum temperature of 1000 °C. This process would eliminate the need for chemicals, such as those used in the case of graphene and graphene oxide composites, that are toxic to the microbial colony. This approach would allow the electrodes to have a structure close to graphite and therefore not have oxygenated compounds on their surface.

## 2. Materials and Methods

### 2.1. Bio-Oil Preparation

Bio-oil production through carbonisation is the result of the thermal decomposition of organic materials in the absence of oxygen. The process involves heating biomass, such as wood or agricultural residues, to high temperatures in a controlled environment, resulting in the production of various by-products, including bio-oil. In this study, coconut shells were used as biomass to produce bio-oil.

During carbonisation, volatile components such as water, tar, and gases are released. These components are the by-products of the thermal decomposition process. The volatile components, including tar, are condensed in a cooler part of the stack. This condensation process results in the formation of bio-oil, a thick, sticky substance. The condensed bio-oil was collected in a separate container through a hole in the curved part of the chimney.

### 2.2. Bio-Pitch Production

After the carbonisation process, the bio-oil in the collection vessel was subjected to heat treatment at 105 °C inside an Erlenmeyer flask with a magnetic stirrer. The heat treatment was carried out until the density reached the value of 1.32 g/cm^3^ which characterises bio-pitch. The final bio-pitch products were then used as raw materials in the production of the carbon-based electrode binder. The properties studied as indicators of good quality of bio-pitch are density and coking index. To determine the coking index, 1 g of bio-pitch was introduced into a hermetically sealed crucible and then treated at 550 °C for two hours in an oven according to the standard test method ASTM D4715-98 [22]. The mass of residual carbon in the crucible was measured. The mass of black residue obtained represents the coking index. These properties are parameters that determine the quality of the bio-pitch. The density and coking value were determined for comparison with the coal-tar pitch commonly used in the carbon anode industry [13].

### 2.3. Electrode Preparation and Characterisation

The charcoal powders used were from *Azadirachta indica* and *Robinia pseudoacacia* wood. These coals were produced by carbonisation between 450 °C and 600 °C. The carboniser used is traditional and the temperature is not fixed. During carbonisation, the temperature is controlled by a thermocouple. The first phase, called the ignition phase, corresponds to a sudden rise in temperature of up to 200 °C. This is a very short phase. The second phase is when part of the biomass burns to supply energy to the rest of the biomass for carbonisation. This is an endothermic phase that takes place between 200 °C and 450 °C in the case of our study. The residue obtained after this phase is not charcoal but rather torrefied wood. Carbonisation proper begins after this phase, at between 450 °C and 600 °C for our biomass. After a comparatively long period of time, the colour of the smoke changes from white to blue. The residue obtained after this phase is charcoal. The temperature drops and carbonisation is stopped to prevent combustion when it starts to rise. After carbonization, the charcoal was crushed and sieved to a size of 50 µm. The powder was mixed with bio-pitch and the resulting paste was shaped into a cylindrical cell 8 mm in diameter and 80 mm in height, which was then compacted under a pressure of 60 MPa. The proportion of bio-pitch in the mixture is 34.55% by mass. After compaction, the samples obtained were demoulded, dried for 72 h, and heat treated under an argon atmosphere at two temperatures (800 °C and 1000 °C) for one hour. The samples were weighed before and after heat treatment with a balance with an accuracy of 0.1. After this heat treatment, the density of the obtained electrodes was measured according to ASTM D5502-00 (2005) [23] and their electrical resistivity was determined using a four-point measurement. The density was calculated using Equations (1) and (2).

(1) $$V\;=\;\frac{\pi hD^{2}}{4}$$

V: anode volume (cm^3^);
h: electrode length (cm);
D: electrode diameter (cm).

(2) $$d=\;\frac{m}{V}\;$$

d: density (g/cm^3^);
m: electrode mass (g).

Two types of electrodes were developed at 800 °C and 1000 °C. One with *Azadirachta indica* charcoal (N800 and N1000) and the other with *Robinia pseudoacacia* charcoal (A800 and A1000). Graphite electrodes extracted from Sunwatt UM1.R20 SIZE D batteries (Hangzhou, China), marked G, were used as the reference (control). The electrical resistivities of the electrodes labelled N800, A800, N1000, A1000, and G were determined using the four-point measurement [14]. The four-point method is used because it is insensitive to the contact resistance between the points and the object. The voltage is measured by placing the sample in the fixture. The vise is closed so that the sample is in contact only with the copper plates soldered to springs which are in turn connected to the current source. The current is varied from 0.01 A to 1A for the electrodes that can tolerate it. This low current is applied to minimise disturbances in the electrodes. Indeed, low current levels ensure that any disturbance in the anode is minimal (as a possibility of local heating due to the Joule effect) [7]. The two terminals of the ammeter that measure the current are connected to the ends of the sample. The length of the sample is divided into three equal distances. The terminals of the voltmeter are placed at the other two internal points so that all the terminals of the two multimeters are aligned. The electrical resistivity was calculated according to the equations below:

(3) $$A=\frac{\pi D^{2}}{4}$$

(4) $$\rho =\frac{UA}{IL}$$

U is the average voltage (V).
A: sample section area (cm^2^);
I: current intensity (A);
L: distance between two points (cm);
D: sample diameter (cm).

Fourier-transform infrared spectroscopy (FTIR) is used to determine the compositional features of the samples and their surface roughness is characterised by atomic force microscopy (AFM). Charge transfer is determined by electrical impedance spectroscopy (EIS). FTIR spectra were measured by a FTIR spectrometer system (PerkinElmer Spectrum 1072) consisting of a DTGS (Deuterated Triglycine Sulphate) detector, a DRIFT accessory, and a microsample. The samples were examined in powder form using KBr as a reference material. The spectra were recorded from 4000 to 500 cm^−1^ by averaging 100 scans at a resolution of 4 cm^−1^. The electrochemical characteristics of the electrodes were measured with the Potentiostat/Galvanostat CS350 CORRTEST in a three-electrode configuration. The working electrode (elaborated electrodes), the Pt electrode as a counter electrode, and a 1 M Ag/AgCl KCl as a reference electrode. The 1 M Na_2_SO_4_ solution was used as a supporting electrolyte during electrochemical impedance spectroscopy (EIS). Electrochemical impedance spectra (EIS) were performed in the frequency range of 10 mHz to 100 kHz to enable detection of comparatively slow phenomena with a signal amplitude of 10 mV to minimise ohmic losses in the electrodes. The surface roughness of the as-developed electrodes was observed using an XE-120 atomic force microscope (Park System Corp., Suwon, Republic of Korea) controlled with XEP software, version 1.8.0, (https://www.ifj.edu.pl/dept/no3/nz34/afm/, accessed on 16 August 2024) for data acquisition and XEI software, version 1.8.0, (https://www.parksystems.com/kr, accessed on 16 August 2024) for image processing and analysis. As a measure of electrode surface roughness, we use Sa= mean roughness, Sq = RMS roughness (grains-wise), Sku = kurtosis, Sz = maximun height, and Sv = maximun pit depth, which provide information on roughness and reactive sites during electrochemical applications.

## 3. Results and Discussion

### 3.1. Bio-Oil Production

The coconut shell carbonisation process yielded 490.5 mL of bio-oil with a density of 1.1 per 3000 g of biomass, i.e., a yield of 17.98% by mass. The density of the bio-oil obtained is consistent with the results in the literature [24]. The yield obtained is lower than that in the literature. This could be explained by the fact that the condensation device used in this study is open and does not allow the recovery of the entire condensable fraction of volatile matter.

### 3.2. Synthesis of Bio-Pitch

#### 3.2.1. Density of the Bio-Pitch

The density of the bio-pitch developed in this study is 1.32 g/cm^3^. This value is in agreement with the results obtained by Ying et al. who characterised three bio-pitches derived from three different hardwood and softwood biomass sources with density values between 1.25 and 1.36 g/cm^3^ [25,26].

#### 3.2.2. Coking Value

Electrical conductivity: The coking of bio-pitch can also affect the electrical conductivity of the carbon electrode. A well-coked coke can have a higher electrical conductivity which is important to ensure the optimum electrochemical performance of the electrode. The bio-pitch obtained was subjected to heat treatment at 550 °C in the absence of oxygen for 2 h to determine the coking index. The results of this analysis give a coking index of 47%. In fact, during the heat treatment process, an appreciable quantity of volatiles, particularly heavy volatiles, escape. This coking index is lower than that of CTP (65.24%), which is considered to be a reference tar. The coking value found in our study is higher than the findings in the literature. For example, Ying Lu et al. [26] found a coking value ranging from 35.27 to 45.30% by mass for softwood and hardwood, respectively. A high coking value is desired to produce an anode with minimal porosity [27]. The high coking value of CTP is attributed to its aromatic nature whereas the bio-pitch encountered contains aliphatic chemical compounds containing oxygen. These oxygen-containing compounds are less stable at elevated temperatures and consequently the bio-pitch showed a low coking value [26]. This high coking value could be explained by a high content of oxygenated compounds in the coconut shells used in our study compared to the values found by Hussein, A. et al. [28].

### 3.3. Preparation and Characterisation of Electrodes

#### 3.3.1. Production of Charcoal

The charcoal used in this study was obtained by carbonising the wood of *Azadirachta indica* and *Robinia pseudoacacia* in a cylindrical carbonizer. For each type of biomass, a mass of 9200 g was introduced into the carbonizer. The mass of charcoal obtained and the carbonisation yield are shown in Table 1.

**Figure 1:**
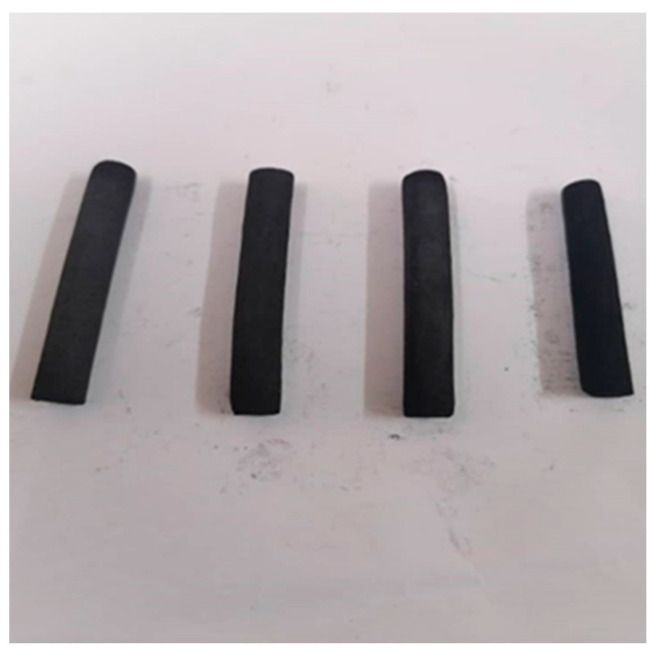
Raw electrodes.

The various coal powders obtained were sieved and then mixed with the bio-pitch The paste obtained was modelled in a cylindrical mould and then pressed. Figure 1 shows the raw electrodes obtained after heat treatment.

#### 3.3.2. Mass Loss and Density After Heat Treatment

Table 2 shows the mass loss of samples of the two different types of electrodes after heat treatment at 800 °C and 1000 °C. It can be seen that, for the two different types, the mass loss of the electrode at 800 °C is less than that at 1000 °C. Higher mass losses indicate greater carbonisation. This result is consistent with that of François Tremblay and A. Platon, for whom mass loss increases with increasing temperature [29,30]. Between 150 and 800 °C, several phenomena occur, such as the flow of pitch into the pores of dry aggregates, the release of light volatile matter contained in the pitch and the release of heavy volatile matter. Between 800 and 1000 °C, there is a significant release of hydrogen and methane [31]. This difference between the mass loss of electrodes heat-treated at 800 °C and 1000 °C could be explained by the release of hydrogen and methane. A comparison of the mass loss of electrodes A800 and N800 on the one hand and A1000 and N1000 on the other show a more pronounced mass loss for electrodes A1000 and A800. This result can be explained by the difference in origin of the charcoal used.

Table 2 displays the density of each developed electrode and that of the graphite in the Leclanche battery. A good anode density is generally considered to be a sign of a good quality anode. The density of a coal coke carbon anode is typically between 1.5 and 1.6 g/cm^3^ [32]. Increasing the heat treatment temperature from 800 °C to 1000 °C allows the particles to break down easily, forming new pores. The additional heat treatment temperature leads to an increase in cell pores, resulting in a decrease in density. Table 2 shows the average density of the electrodes produced at 800 °C and 1000 °C. Table 2 shows a significant drop in the density of electrodes as the temperature increases. This is due to the carbonisation process, which eliminates the other competing elements of the carbon, thus reducing the density of the carbon electrode. At this stage, carbon is not resistant enough and requires activation to become stronger, which leads to the formation of more pores. During thermal treatment, the carbon is scratched by the reaction of CO_2_ gas with its surface. Oxygen comes from the elimination of elements during treatment. This process leads to the production of new pores and to the development of preexisting pores [19]. The very high treatment temperature creates more vaporised compound, which reduces the mass and density of the electrode. The data in Table 2 show that the highest density drop occurred when the processing temperature was 1000 °C. This increase in temperature brought other elements such as oxygen and hydrogen to the surface of the carbon electrode to undergo more evaporation [20] and to produce more pores and finally leading to the lowest densities. These results show that the variations in density in the biomass-based carbon electrode, depending on the carbonization temperature were influenced by cellulose, hemicellulose, and lignin. The density of the electrodes produced is lower than that of G. This could be attributed to the size of the charcoal pores, which are probably too small to be completely filled with pitch during mixing. This phenomenon was observed in the study by Hussein et al. [20]. The low density of the developed electrodes compared to that of G suggests that their porosity is higher than that of G. We will continue our study by determining electrical resistivity, which is a very important parameter to assess the quality of the electrodes.

#### 3.3.3. Electrical Resistivity

Electrical resistivity is considered to be an essential attribute for the training of anodes in P-MFCs with a favourable electron transfer rate value through the electrode [1]. The curves giving electrical resistance and the table summarising the resistivity of the electrodes were developed according to the four-point method.

**Figure 2:**
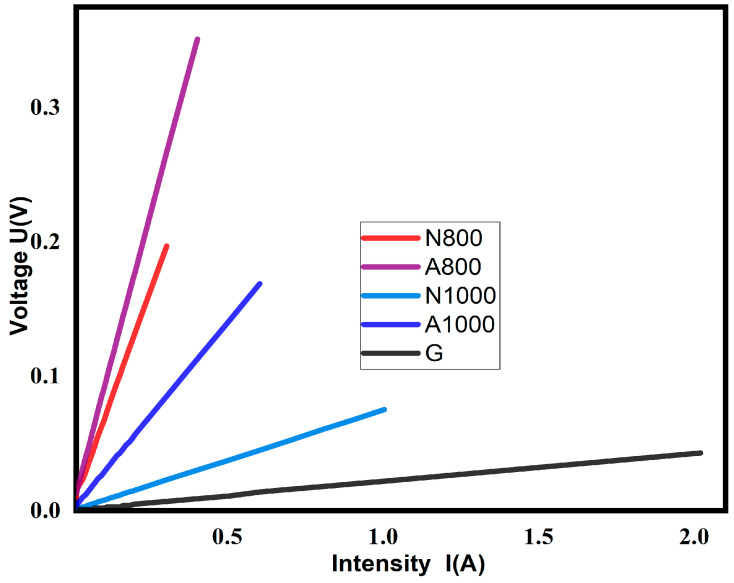
Electrical resistivity of N800, A800, N1000, A1000, and G.

The curves of Figure 2 seem to have a similar appearance. They represent the *U* = *f* (*i*) characteristics of the device (voltage as a function of the current). The slope of each curve represents the value of the resistance according to the law of ohm. The linearity of the curves suggests a good distribution of the bio-pitch. This could be due to the homogeneity of the anodes produced. Table 3 presents the electrical resistivity of the N800, A800, N1000, and A1000 electrodes thermally treated at 800 °C and 1000 °C.

The increase in temperature from 800 °C to 1000 °C was accompanied by a decrease in resistivity of almost four orders of magnitude for *Robinia pseudoacacia* electrodes and almost nine orders of magnitude for the electrodes of the *Azadirachta indica* electrode. Increasing the heat treatment temperature from 800 °C to 1000 °C reduced the resistivity by 68.75% for *Robinia pseudoacacia* electrodes and by 88.79% for *Azadirachta indica* electrodes. This result is similar to that obtained by Kazuhiro in 2003, who found a 43% decrease in resistivity after heat treatment [6]. The decrease in electrical resistivity with the increase in temperature would be attributed to the growth and better alignment of graphite structures. The growth of conductive surface domains and their crystallites is favoured by the loss of heteroatoms dependent on thermal treatment temperature, such as nitrogen, oxygen, and hydrogen [33], which disturb the conductive structure of the carbon and subsequent mechanisms of the reticulation, alignment, and fusion of carbon structures [34]. These results collaborate those of Julian Borowec et al. in 2023 [35]. Based on the heat treatment temperature and its influence on electrical resistivity, the results clearly show that an increase in the heat treatment temperature promotes lower values of electrical resistivity which can be attributed to different underlying processes, such as the increase in the ash content of coal with increasing treatment temperature [19], the progressive change of carbon structures mainly from σ-bonds and sp^3^-state carbon (in the biomass precursor) to sp^2^-state conjugated carbon during carbonisation, the decrease in acidic functionalities on the carbon surface after 800–1000 °C, and the reorganisation of amorphous carbon structures to crystalline structures from 1000 °C to 2500 °C [20]. Figure 2 shows the resistivity of A1000 and N1000. The values reveal a similar trend to those of A800 and N800, but A1000 has a higher resistivity than N1000. Similarly, the resistivity of A800 is higher than that of N800. This result is in agreement with all the results found previously in our work. Indeed, the A1000 electrode shows a greater mass loss than the N1000, which could be related to the highly volatile content of A1000. The same is true for A800 and N800, where N800 has a lower resistivity than A800. The resistivity of A1000 is lower than that of N1000 while its density is higher than that of N1000. This result seems to contradict the statement of Yadav, A.K. [2]. According to Yadav, a high-density electrode should have a higher resistivity than a low-density electrode. Density would therefore not be a necessary and sufficient parameter for a good electrode quality. This result is in agreement with that found by Kazuhiro Mochidzuki in 2003. Kazuhiro found 0.35 Ω.cm as the resistivity of a powder with a density of 0.78 g/mL, which is lower than the value of 0.41 Ω.cm associated with the same mesh material of 0.61 g/mL [2]. The developed electrodes show good electrical performance compared to that of Coutinho [8] who used eucalyptus wood as a source for the production of pitch and coke [4], and to Kiminaite Leva in 2022, who found 1.22 10^−2^ Ω.m [1]. The highest electrical resistivity value in our study is more than 40 times lower than that found in the study by Assem in 2022 using fossil pitch [10]. Of all the electrodes developed, N1000 has the lowest resistivity. However, the electrical resistivity of N1000 is 12.10% higher than that of G. The low electrical resistivity of G compared to N1000 could be explained by their relative densities. Electrical resistivity increases with increasing pore size (low density) [27]. FTIR characterisation showed a higher degree of graphitisation compared with N1000. This could justify the low resistivity of G compared with N1000. Indeed, a high level of graphitisation is a sign of good electrical conductivity [1]. The advantage it has over G is that its production method is environmentally friendly and cheaper. Electronic transfer depends not only on the resistivity but also on the surface morphology. Electrochemical impedance spectroscopy of the electrodes enables the evaluation of activation, charge transfer, and diffusion phenomena.

#### 3.3.4. Fourier-Transform Infrared Spectroscopy (FTIR)

**Figure 3:**
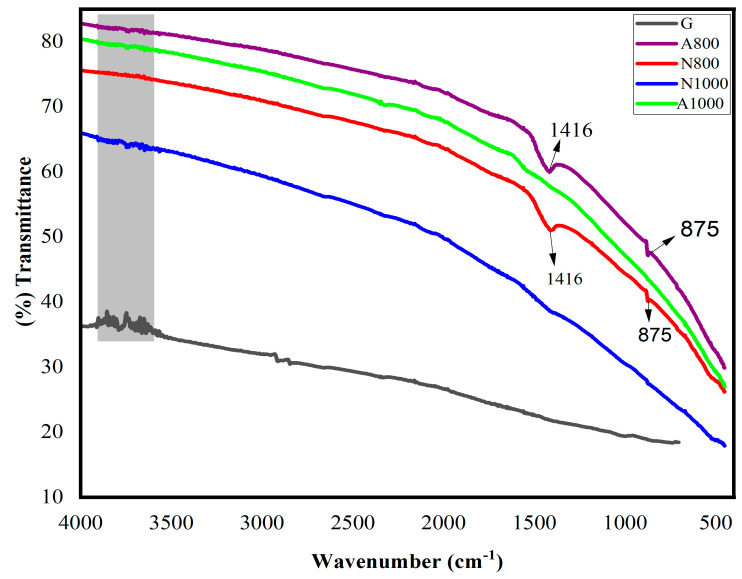
FTIR spectra of the electrodes and graphite of the Leclanche battery.

The FTIR spectrum was used to identify the main functional groups in the structure. The FTIR spectra of the electrodes prepared at 800–1000 °C are shown in Figure 3. For comparison, the spectrum of graphite from the Leclanche battery is also shown. Small differences in the positions and shapes of the bands, marked by the band area, probably represent particle size and scattering effects rather than changes in electron mobility. These effects can hardly be eliminated from the drift spectra of carbonised coals and graphite. These effects are also observed in the studies of Kazuhiro Mochidzuki in 2003 [36].

As the heat treatment temperature increases from 800 °C to 1000 °C, the overall intensity of the infrared absorption spectrum increases. Electrodes N1000 and A1000 have a higher intensity of absorption in the infrared spectrum than electrodes N800 and A800, respectively. At 800 °C, A800 and N800 show a characteristic peak at 1416 cm^−1^ attributed to the C(tetrahedral)-H deformation vibration and a characteristic peak at 875 cm^−1^ attributed to the 1,2,4-trisubstituted C(trigonal)-H(aromatic) deformation vibration. This weak absorption of CH at 875 cm^−1^ is attributed to isolated hydrogen atoms on the edges of the condensed aromatic sheets [36]. These results corroborate studies by Friel et al. [37]. These peaks obtained from A800 and N800 could be due to the residue of aromatic hydrocarbons and methane generally contained in bio-pitch. Between 500 and 900 °C, significant amounts of hydrogen and methane are released by polymerisation reactions of the large molecules contained in the pitch during heat treatment of the electrodes [38]. The graphite electrode has no characteristic infrared band in the region studied. This behaviour of the graphite electrode could be due to its insensitivity to the long range order during graphitisation [39]. The suppression of the absorption spectra of the N1000 and A1000 electrodes can be explained by the fact that the side chains and heteroatoms are released in this temperature range, leading to a more graphitic structure. This result shows that increasing the temperature leads to the graphitisation of the structure of the electrodes developed. A high level of graphitisation is a sign of good electrical conductivity [1]. The decrease in the electrical resistivity of A1000 by nearly four orders of magnitude and that of N1000 by nearly nine orders of magnitude compared to that of A800 and N800, respectively, could be explained by the more graphitic structure of A1000 and N1000 compared to A800 and N800, respectively. The N1000 and A1000 electrodes show very similar spectra to each other and to the spectrum of G. The functional groups obtained for the electrodes treated at 800 °C would be vaporised in the form of matter at 1000 °C. This result collaborates the study of Senthilkumar, K in 2023 [40]. N1000 has a similar spectrum and an absorption intensity more G than A1000. This result shows the more graphitic structure of N1000 than any of the electrodes developed.

#### 3.3.5. Electrochemical Response

**Figure 4:**
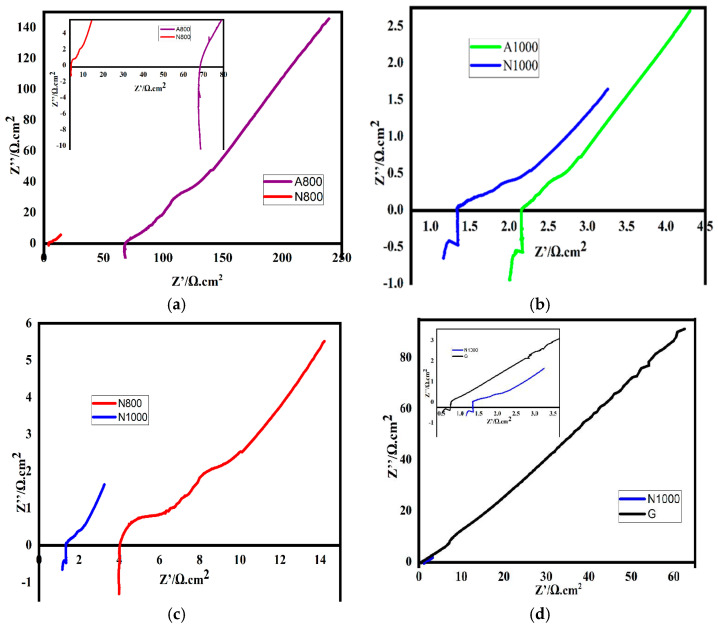
(**a**) Nyquist curves for N800 and A800; (**b**) Nyquist curves for N1000 and A1000; (**c**) Nyquist curves for N1000 and N800; (**d**) Nyquist curves for N1000 and G.

Figure 4a–d have a similar appearance, suggesting that they reproduce the same phenomenon. The drop in the data at the bottom of the real axis can be explained by parasitic capacitance due to electromagnetic interference from the cables. These phenomena generally occur at high frequencies and low impedances, as observed in our study. Indeed, the alternating current flowing in the wires carrying the current produces a magnetic field that couples to the wires from which the potential is measured, leading to unwanted voltages that can cause capacitance errors. This same phenomenon occurred when measuring the electrochemical impedance spectroscopy of cement paste electrodes in Aldo F. SOSA Gallardo’s study in 2021 [6]. The absence of a semicircle or the bioelectrochemical reaction resistance of the electrodes in each of these figures means that the charge transfer resistance is zero or almost zero. This means that electrons are well transferred. The presence of a line represents a significant capacitive effect. Figure 4a,b on the one hand and Figure 4c,d on the other hand, respectively, show the influence of the material and the heat treatment temperature on the electrochemical impedance spectrum. These curves reflect the characteristics of the capacitive behaviour of a porous electrode, as in the case of O. Gharbi in 2020 [10], where the shape of these curves reflects a limited diffusion or mass transfer process at the electrode–electrolyte interface. For these electrodes, high-frequency EIS measurements are significantly affected by the choice of electrode material and the heat treatment temperature. These differences are notable by the intersection of the curve with the real axis, and the deviation from the ideal behaviour of the capacitor and the ohmic resistance range defined by the imaginary part. In Figure 4b,c, A800 and A1000 have a higher electrolytic resistance (Re) than N800 and N1000. By definition *R_e_* = *R_s_* + *R_fil_* + *R_elec_* with *R_s_* the solution resistance, *R_fil_* the contact wire resistance and, *R_elec_* the electrode resistance [41]. As a result, the electrode resistance of A800 and A1000 is higher than that of N800 and N1000, respectively (Table 4). The shift in electrolyte resistance towards higher values of A800 compared to N800 and A1000 compared to N1000 is thought to be due to the small pore size of A800 compared to N800 and the small pore size of A1000 compared to N1000.

Porosity: The manner in which bio-pitch cokes can also influence the porosity of the electrode. Adequate porosity is important to allow the flow of gases and electrolytes, which is essential in some electrochemical applications. The small pore size makes it difficult for the electrolyte to penetrate into the porous structure of the electrode [9].

Following a similar reasoning for Figure 4d, the N1000 electrode performed better than all the developed electrodes. This result is in agreement with our previous results on mass loss and resistivity. Table 4, showing the electrolyte resistance and n (the deviation from the ideal behaviour of the capacitor), also confirms this result. The value of the capacitive effects of G is considerably lower than that of the N1000. This decrease can be explained by the decrease in electrode resistance. This result is consistent with the findings of Aldo. F. Sosa Gallardo [6]. According to the studies of M. Guo and P. Diao [42], the imaginary part describes the losses in the electrodes. In Figure 4d, the Nyquist curve for G has a higher imaginary part than that for the N1000 electrode. This suggests that this behaviour is related to a lower electron transfer rate for the graphite electrode compared to the N1000 electrode. A linear line with a high imaginary part suggests that electrons are poorly transported [10]. The N1000 electrode would therefore have a rougher surface favourable to electron transfer than G. This result is in agreement with that found by Linghan Lan in 2019 [11]. The result according to which the lower resistance to high-frequency interception with the real axis does not present a lower resistance to charge transfer. This phenomenon is facilitated in this study by the rough surface of the electrode. These results clearly confirm the better electrochemical quality of N1000 compared to G.

#### 3.3.6. Morphological Characterisation by Atomic Force Microscopy (AFM)

**Figure 5:**
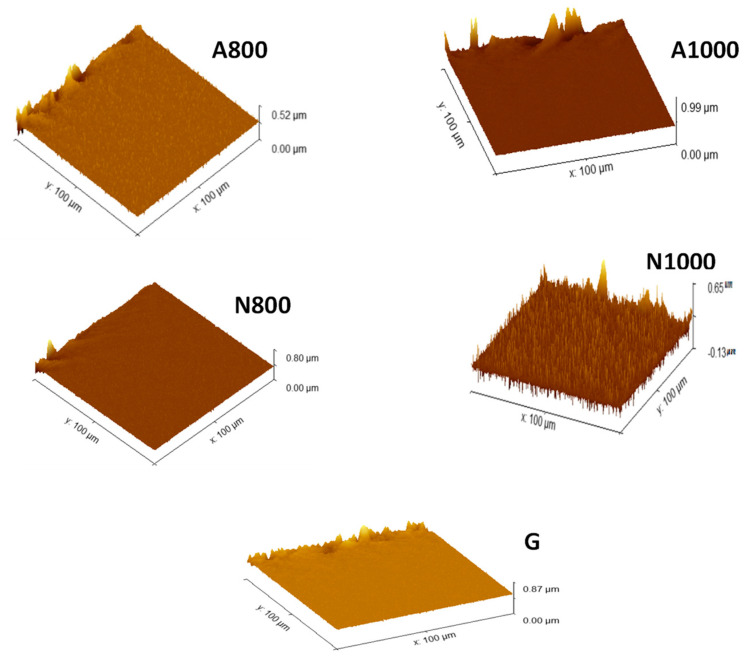
3D images of the elaborate electrodes.

Figure 5 shows the AFM images of the electrodes with the heat treatment process temperature ranging from 800 °C to 1000 °C and the roughness parameters are summarised in Table 5. Comparison of the AFM images shows that the heat treatment causes an increase in roughness. The average roughness (Sa) of A800 increases from 59.904 nm to 63.251 nm for A1000 and the average roughness (Sa) of N800 increases from 47.0276 nm to 69.036 nm for N1000 (Table 5). These results show that the aliphatic side chains and heteroatoms are released in this temperature range, resulting in the aromatisation of the entire structure. This increase in roughness could be explained by the degradation of a major chemical component, namely lignin. During the heat treatment, the degradation of volatile materials (cellulose and hemicellulose) occurs between 300 and 500 °C, and for lignin it occurs at higher temperatures [43]. Beyond 800 °C, it is lignin that begins to decompose by a reorganisation of carbon atoms into a graphitic-like structure, releasing a large amount of gas or volatile materials such as H_2_ [44]. During this process, a porous carbon skeleton is formed by deformed aromatic strip/sheet structures, which resembles a morphology with wrinkled, porous, and interconnected network microstructures [45]. As a result, the original surface could be deformed, and the surface roughness could be enhanced. N1000 has a rougher surface than A1000. The average roughness (Sa) of N1000 is higher than that of G. The surface of N1000 has more folds and wrinkles (Figure 5), while a comparatively smooth surface is the characteristic of G. The variation of roughness is consistent with the results of roughness parameter values (Table 5). The AFM results are in agreement with the EIS test, which shows that N1000 has more active reactive sites than G despite its lower electrical resistivity compared to G. On the other hand, the AFM confirms the FTIR results according to which N1000 has a more pronounced graphitic structure than all the electrodes developed. In short, N1000 has a roughness that correlated well with its electrical resistivity so that the bacterial colony would grow comfortably in the P-MFC and a sufficient charge transfer rate could be achieved.

## 4. Conclusions

The present study highlights the synthesis of electrodes based on charcoal and bio-pitch, two components from a renewable, clean, and sustainable source. Charcoal replaces commercial petroleum coke and bio-pitch replaces coal-tar pitch. Both are capable of reducing emissions in industrial processes which use petroleum coke and coal-tar pitch for electrode synthesis. This approach focuses on a new method for electrode preparation. The density and coking value of bio-pitch make it possible to substitute fossil pitch with bio-pitch. Several characterisations were carried out to evaluate the excellence of the synthesised material. The increase in the heat treatment temperature improved the performance of the electrodes in terms of electrical resistivity, highlighted by the four-point method. FTIR shows that the spectrum of N1000 is preferable compared to the spectrum of G and of the other prepared electrodes. The EIS results demonstrated that there are more ions found on the surface of N1000 compared to G. This means that the N1000 prepared herein has more reactive sites that are well correlated with its electrical resistivity. All the electrodes in this study have been intensively studied by atomic force microscopy (AFM) to evaluate the resulting morphological evolution of the surface. The surface roughness was found to increase with increasing temperature. AFM shows that the mobility of the ions depends on the electrical resistivity, which should correlate with the roughness. Next, we will evaluate the performance of bioelectrodes produced by a plant-based microbial fuel cell using lemongrass.

## Figures and Tables

**Table 1:** Yield of coke obtained in relation to carbonised wood.

Type of Wood	Mass of Carbonized Wood (g)	Mass of Coal Obtained (g)	Coal Yield (%)
*Azadirachta indica*	9200	4118	44.76
*Robinia pseudoacacia*	9200	3856	41.91

**Table 2:** Showing mass loss at different annealing temperatures.

Annealing Temperature (°C)	800		1000		-
Type of charcoal	(N800)	(A800)	(N1000)	(A1000)	Leclanche battery electrode
Mass loss after annealing (%)	37.20	40.87	47.45	56.77	-
Density (g/cm^3^)	0.96	0.91	0.81	0.80	1.80

**Table 3:** Electrical resistivity of the different electrodes.

Electrode	A800	N800	A1000	N1000	G
ρ in (μΩm)	1.60	1.16	0.5	0.13	0.05

**Table 4:** Characteristics of selected samples.

Sample	$R_{e}\;\mathrm{in}\;Ω/{cm}^{2}$	*n*
A800	69.570	0.124
A1000	2.014	0.280
N800	3.985	0.201
N1000	1.165	0.327
G	0.511	0.511

**Table 5:** Surface profile parameters for the engineered electrodes.

Parameters	A800	N800	A1000	N1000	G
Sa (nm)	59.904	47.0276	63.251	69.036	64.450
Sq (nm)	107.053	96.0350	113.155	127.235	123.073
Sku	8.36320	13.6314	9.04303	7.45238	8.30044
Sz (µm)	1.18848	1.14395	1.70674	1.73661	1.24585
Sv (µm)	0.53463	0.46902	0.91364	0.92079	0.62698

Sa = mean roughness, Sq = RMS roughness (grain-wise), Sku = kurtosis, Sz = maximum height, Sv = maximum pit depth, A800 = *Robinia pseudoacacia* electrode treated at 800 °C, N800 = *Azadirachta indica* electrode treated at 800 °C, A1000 = *Robinia pseudoacacia* electrode treated at 1000 °C, N1000 = electro *Azadirachta indica* de treated at 1000 °C and G = graphite electrode of the Leclanche battery (reference).

## Data Availability

The original contributions presented in the study are included in the article, further inquiries can be directed to the corresponding author.

## References

[1] Kiminaitė I., Lisauskas A., Striūgas N., Kryževičius Ž. (2022). Fabrication and characterization of environmentally friendly biochar anode. Energies.

[2] Yadav A.K., Dash P., Mohanty A., Abbassi R., Mishra B.K. (2012). Performance assessment of innovative constructed wetland-microbial fuel cell for electricity production and dye removal. Ecol. Eng..

[3] Guan C.Y., Tseng Y.H., Tsang D.C.W., Hu A., Yu C.P. (2019). Wetland plant microbial fuel cells for remediation of hexavalent chromium contaminated soils and electricity production. J. Hazard. Mater..

[4] Arends J.B.A., Speeckaert J., Blondeel E., De Vrieze J., Boeckx P., Verstraete W., Rabaey K., Boon N. (2014). Greenhouse gas emissions from rice microcosms amended with a plant microbial fuel cell. Appl. Microbiol. Biotechnol..

[5] Sonu K., Sogani M., Syed Z., Dongre A., Sharma G. (2021). Improved decolorization of dye wastewater and enhanced power output in the electrically stacked microbial fuel cells with H_2_O_2_ modified corncob anodes. Environ. Prog. Sustain. Energy.

[6] Gallardo A.F.S., Provis J.L. (2021). Electrochemical cell design and impedance spectroscopy of cement hydration. J. Mater. Sci..

[7] Kocaefe Y., Kocaefe D., Bhattacharyay D. (2015). Quality Control via Electrical Resistivity Measurement of Industrial Anodes. Light Met..

[8] Coutinho A.R., Rocha J.D., Luengo C.A. (2000). Preparing and characterizing biocarbon electrodes. Fuel Process. Technol..

[9] Hussein A., Wang Z., Ratvik A.P., Grande T., Alamdari H. (2022). Electrochemical Performance of Carbon Anodes Made of Bio-pitch as a Binder. Metall. Mater. Trans. B Process Metall. Mater. Process. Sci..

[10] Gharbi O., Tran M.T.T., Tribollet B., Turmine M., Vivier V. (2020). Revisiting cyclic voltammetry and electrochemical impedance spectroscopy analysis for capacitance measurements. Electrochim. Acta.

[11] Lan L., Li J., Feng Q., Zhang L., Fu Q., Zhu X., Liao Q. (2019). Enhanced current production of the anode modified by microalgae derived nitrogen-rich biocarbon for microbial fuel cells ScienceDirect Enhanced current production of the anode modified by microalgae derived nitrogen-rich biocarbon for microbial fuel cells. Int. J. Hydrogen Energy.

[12] Sekeri S.H., Ibrahim M.N.M., Umar K., Yaqoob A.A., Azmi M.N., Hussin M.H., Othman M.B.H., Malik M.F.I.A. (2020). Preparation and characterization of nanosized lignin from oil palm (*Elaeis guineensis*) biomass as a novel emulsifying agent. Int. J. Biol. Macromol..

[13] Yaqoob A.A., Ibrahim M.N.M., Umar K. (2021). Biomass-derived composite anode electrode: Synthesis, characterizations, and application in microbial fuel cells (MFCs). J. Environ. Chem. Eng..

[14] Yang W., Li J., Lan L., Li Z., Wei W., Lu J.E., Chen S. (2019). Facile Synthesis of Fe/N/S-Doped Carbon Tubes as High-Performance Cathode and Anode for Microbial Fuel Cells. ChemCatChem.

[15] Yaqoob A.A., Ibrahim M.N.M., Yaakop A.S., Rafatullah M. (2022). Utilization of biomass-derived electrodes: A journey toward the high performance of microbial fuel cells. Appl. Water Sci..

[16] Yaqoob A.A., Ibrahim M.N.M., Rafatullah M., Chua Y.S., Ahmad A., Umar K. (2020). Recent advances in anodes for microbial fuel cells: An overview. Materials.

[17] Yang W., Chen S. (2020). Biomass-Derived Carbon for Electrode Fabrication in Microbial Fuel Cells: A Review. Ind. Eng. Chem. Res..

[18] Choi K.C., Lee E.K., Choi S.Y., Park S.J. (2003). Electrical and Physical Properties of Carbonized Charcoals. Polymer.

[19] Sonu K., Sogani M., Syed Z., Rajvanshi J., Pandey S.C. (2024). Performance evaluation of Epipremnum aureum plant-based microbial fuel cell using composite anode made up of carbonized corncob and carbon rod. Biomass Convers. Biorefinery.

[20] Hussein A., Fafard M., Ziegler D., Alamdari H. (2017). Effects of charcoal addition on the properties of carbon anodes. Metals.

[21] Amara B. (2022). Utilisation du Biocharbon dans la Production des Anodes en Carbone. Ph.D. Thesis.

[22] (2022). Standard Test Method for Coking Value of Tar and Pitch (Alcan).

[23] (2005). Standard Test Method for Apparent Density by Physical Measurements of Manufactured Anode and Cathode Carbon Used by the Aluminum Industry.

[24] Silva F.T.M., Ataíde C.H. (2019). Valorization of eucalyptus urograndis wood via carbonization: Product yields and characterization. Energy.

[25] Thieblesson L.M., Djomo A.S., Kouassi K.A. (2021). Caractérisation physique et hygrothermique des matières premières en vrac (granules de papier, ouate de cellulose et fibre de bois) pour l’ élaboration d’ éco-matériaux. Afr. Sci..

[26] Lu Y., Mollaabbasi R., Picard D., Ziegler D., Alamdari H. (2019). Physical and chemical characterization of bio-pitch as a potential binder for anode. Light Metals 2019.

[27] Amrani S., Kocaefe D., Kocaefe Y., Bhattacharyay D., Bouazara M., Côté J. (2021). Effect of carbon anode production parameters on anode cracking. SN Appl. Sci..

[28] Hussein A., Lu Y., Mollaabbasi R., Tessier J., Alamdari H. (2020). Bio-pitch as a binder in carbon anodes for aluminum production: Bio-pitch properties and its interaction with coke particles. Fuel.

[29] Rocha J.D., Coutinho A.R., Luengo C.A. (2002). Biopitch produced from eucalyptus wood pyrolysis liquids as a renewable binder for carbon electrode manufacture. Braz. J. Chem. Eng..

[30] Doat J. (1977). Le pouvoir calorifique des bois tropicaux. Revve Bois For. Trop..

[31] Prauchner M.J., Pasa V.M.D., Otani S., Otani C. (2005). Biopitch-based general purpose carbon fibers: Processing and properties. Carbon N. Y..

[32] Kocaefe Y., Charette A., Bui R.T. Heat transfer modelling of the combustion chamber of a sidewell furnace. Proceedings of the 9th International Conference on Heat Transfer, Fluid Mechanics and Thermodynamics.

[33] Kretzschmar A., Selmert V., Weinrich H., Kungl H., Tempel H., Eichel R.A. (2020). Tailored Gas Adsorption Properties of Electrospun Carbon Nanofibers for Gas Separation and Storage. ChemSusChem.

[34] Park J., Kretzschmar A., Selmert V., Camara O., Kungl H., Tempel H., Basak S., Eichel R.A. (2021). Structural Study of Polyacrylonitrile-Based Carbon Nanofibers for Understanding Gas Adsorption. ACS Appl. Mater. Interfaces.

[35] Borowec J., Selmert V., Kretzschmar A., Fries K., Schierholz R., Kungl H., Eichel R., Tempel H., Hausen F. (2023). Carbonization-Temperature-Dependent Electrical Properties of Carbon Nanofibers—From Nanoscale to Macroscale. Adv. Mater..

[36] Mochidzuki K., Soutric F., Tadokoro K., Antal M.J. (2003). Electrical and physical properties of carbonized coals. Polymer.

[37] Friel J.J., Mehta S., Follweiler D.M. (1982). Electron Optical and IR Spectroscopic Investigation of Coal Carbonization. Coal and Coal Products: Analytical Characterization Techniques.

[38] Amrani S., Kocaefe D., Kocaefe Y., Bhattacharyay D., Bouazara M., Coulombe P. (2017). Evolution of anode properties during baking. IJISET-Int. J. Innov. Sci. Eng. Technol..

[39] Mapelli C., Castiglioni C., Meroni E., Zerbi G. (1999). Graphite and graphitic compounds: Vibrational spectra from oligomers to real materials. J. Mol. Struct..

[40] Senthilkumar K., Naveenkumar M. (2023). Enhanced performance study of microbial fuel cell using waste biomass-derived carbon electrode. Biomass Convers. Biorefinery.

[41] El Mekawy A., Hegab H.M., Mohanakrishna G., Pant D., Wang H. (2018). Integrated Bioelectrochemical Platforms.

[42] Diao P., Guo M., Jiang D., Jia Z., Cui X., Gu D., Zhong B. (2000). Fractional coverage of defects in self-assembled thiol monolayers on gold. J. Electroanal. Chem..

[43] Boraah N., Chakma S., Kaushal P. (2023). Optimum features of wood-based biochars: A characterization study. J. Environ. Chem. Eng..

[44] Wei X., Wei J.S., Li Y., Zou H. (2019). Robust hierarchically interconnected porous carbons derived from discarded Rhus typhina fruits for ultrahigh capacitive performance supercapacitors. J. Power Sources.

[45] Xian F., Gao L., Zhang Z., Zhang H., Dong S., Cui G. (2019). N, P dual-doped multi-wrinkled nanosheets prepared from the egg crude lecithin as the efficient metal-free electrocatalyst for oxygen reduction reaction. Appl. Surf. Sci..

